# Defining Disease Phenotypes Using National Linked Electronic Health Records: A Case Study of Atrial Fibrillation

**DOI:** 10.1371/journal.pone.0110900

**Published:** 2014-11-04

**Authors:** Katherine I. Morley, Joshua Wallace, Spiros C. Denaxas, Ross J. Hunter, Riyaz S. Patel, Pablo Perel, Anoop D. Shah, Adam D. Timmis, Richard J. Schilling, Harry Hemingway

**Affiliations:** 1 Farr Institute of Health Informatics Research, University College London, London, United Kingdom, and Clinical Epidemiology, Department of Epidemiology and Public Health, University College London, London, United Kingdom; 2 Institute of Psychiatry, Psychology and Neuroscience, King's College London, London, United Kingdom; 3 Melbourne School of Global and Population Health, The University of Melbourne, Melbourne, Australia; 4 Barts NIHR Biomedical Research Unit, Queen Mary University London, London, United Kingdom; 5 The Heart Hospital, University College London NHS Trust, London, United Kingdom; 6 London School of Hygiene and Tropical Medicine, London, United Kingdom; Innsbruck Medical University, Austria

## Abstract

**Background:**

National electronic health records (EHR) are increasingly used for research but identifying disease cases is challenging due to differences in information captured between sources (e.g. primary and secondary care). Our objective was to provide a transparent, reproducible model for integrating these data using atrial fibrillation (AF), a chronic condition diagnosed and managed in multiple ways in different healthcare settings, as a case study.

**Methods:**

Potentially relevant codes for AF screening, diagnosis, and management were identified in four coding systems: Read (primary care diagnoses and procedures), British National Formulary (BNF; primary care prescriptions), ICD-10 (secondary care diagnoses) and OPCS-4 (secondary care procedures). From these we developed a phenotype algorithm via expert review and analysis of linked EHR data from 1998 to 2010 for a cohort of 2.14 million UK patients aged ≥30 years. The cohort was also used to evaluate the phenotype by examining associations between incident AF and known risk factors.

**Results:**

The phenotype algorithm incorporated 286 codes: 201 Read, 63 BNF, 18 ICD-10, and four OPCS-4. Incident AF diagnoses were recorded for 72,793 patients, but only 39.6% (N = 28,795) were recorded in primary care and secondary care. An additional 7,468 potential cases were inferred from data on treatment and pre-existing conditions. The proportion of cases identified from each source differed by diagnosis age; inferred diagnoses contributed a greater proportion of younger cases (≤60 years), while older patients (≥80 years) were mainly diagnosed in SC. Associations of risk factors (hypertension, myocardial infarction, heart failure) with incident AF defined using different EHR sources were comparable in magnitude to those from traditional consented cohorts.

**Conclusions:**

A single EHR source is not sufficient to identify all patients, nor will it provide a representative sample. Combining multiple data sources and integrating information on treatment and comorbid conditions can substantially improve case identification.

## Introduction

One of the major challenges presented by the increasing use of electronic health record (EHR) data for research is the development of strategies for reliably identifying disease cases [1]–[4]. Hripcsak and Albers [5] argue that in order to improve the extraction of disease information from this type of data:

*…[W]e need a better understanding of the EHR. The EHR is not a direct reflection of the patient and physiology, but a reflection of the recording process inherent in healthcare with noise and feedback loops. We must study the EHR as an object in itself, as if it were a natural system.*(p. 119)

This recommendation is particularly relevant to identification of chronic conditions in which patients may have multiple interactions with primary and secondary care, and undergo assessments and diagnostic tests, before ultimately receiving a diagnosis. Even after diagnosis, patients may receive follow-up care such as monitoring, prescriptions, or other medical interventions [6]. Consequently, one EHR data source rarely covers the full patient journey; usually data from different record sources (e.g. primary, secondary, and tertiary care; medication prescription and dispensing; mortality data) must be integrated to obtain a complete picture [7]. However, these data also encompass variation in patient measurement that may be context-dependent and thus effective integration requires an exploration of what is recorded in the EHR in relation to a particular condition, and how this compares to expectations based upon guidelines and preconceptions about clinical practice [4], [8], [9].

To highlight the challenges and complexities of identifying onset of a chronic condition in linked national EHR data, and how these can inform the development of strategies for identifying patients, we present a case study of atrial fibrillation (AF) using national linked EHR and administrative health data from the English National Health Service (NHS). AF is the most common cardiac arrhythmia, associated with increased risk of stroke, heart failure (HF), and premature mortality [10], [11.]. It presents many important challenges that may be encountered when developing strategies for case identification, or phenotypes, in EHR data including variability in symptoms and signs, different coding strategies and treatment options, and changes in clinical practice (for more in-depth discussion see [12]).

### Clinical context of atrial fibrillation

Onset of AF often precedes diagnosis considerably; patients may be asymptomatic or experience paroxysmal AF (characterized by irregular, sudden symptoms) and clinical signs, such as irregular pulse, may be episodic. AF may also be diagnosed when a patient is admitted to hospital for another, potentially unrelated, condition. UK diagnostic guidelines and those from the European Society of Cardiology (ESC) recommend pulse palpation followed by an electrocardiogram if an irregular pulse is detected [13], [14]. Opportunistic screening of patients over the age of 65 is recommended by the ESC, but not by North American organisations [15].

Confirming an AF diagnosis does not necessarily simplify documentation as recording and treatment may differ between primary and secondary care, which use different coding systems with different levels of granularity. Read codes, a subset of the Systematic Nomenclature Of Medicine - Clinical Terms (SNOMED-CT) clinical terminology, are used in primary care and permit specification of disease subtypes and differentiation of AF from atrial flutter. In contrast, the International Classification of Disease – 10^th^ revision (ICD-10) terminology used in secondary care has one term for all categories. Treatment varies between patients depending upon symptoms, age and other clinical characteristics, and clinical context. Currently, most patients receive anti-thrombotic treatment to reduce stroke risk, although drugs for rate or rhythm control, and procedures such as cardioversion or catheter ablation, may also be used [14].

AF diagnostic and treatment practices have changed substantially over the last 10–15 years. This is due to increasing awareness of AF and recognition that, at least in the UK, it is more likely to be subject to under, rather than over, diagnosis [16], [17]. Policy initiatives have been introduced in the UK to address this including: the 2004 Quality and Outcomes Framework (QOF), which financially rewards general practitioners for implementing treatment plans for chronic conditions, including AF [18]; the 2006 UK National Institute for Health and Care Excellence guidelines for AF diagnosis and management [19]; the English NHS Commissioning for Quality and Innovation (CQUIN) scheme, introduced in 2009 to provide financial incentives for quality improvements. Thus there may be temporal differences in coding practices for AF.

### Identification of patients with atrial fibrillation

A consistent approach to integrating EHR and administrative health data to identify AF will facilitate transparent and reproducible research, but currently no universal method exists. Previous UK EHR studies have focused on primary care [20]–[23], but other studies used secondary care data. We reviewed research on AF risk factors and found substantial variation in the data sources used to identify AF cases; 21 of 27 studies identified used EHR data, with two using primary care [24], [25], 15 using secondary care [26]–[40], and four using both [41]–[44]. However, many researchers are developing strategies for integrating EHR data for research and defining EHR phenotypes [2], [45]–[51]. The USA-based eMERGE Consortium have developed an AF phenotype algorithm [52], but this was created for data from nine health care providers actively participating in research and focuses on clinical notes and electrocardiogram impressions. As these data are not available on a large scale to researchers in the UK, and elsewhere, using data from nationalised health services, our aims were to develop an understanding of EHR data relating to AF, and to use this to develop a phenotype algorithm applicable to linked, nationally collected data.

Thus we describe the development of the ClinicAl disease research using Linked Bespoke studies and Electronic Records (CALIBER) AF phenotype and use this to demonstrate how exploration of recording patterns in multiple data sources can inform the development of disease case identification strategies for EHR data. We investigated whether EHR data beyond diagnosis codes could be leveraged to refine date of disease onset; whether cases could be inferred on the basis of medical treatment; and whether changes in health care policy may have affected data collection. We evaluated the face validity of the phenotype by testing for associations with known risk factors. The strategies used and lessons learned are broadly applicable to all EHR phenotype development, particularly where the aim is to identify disease cases for longitudinal research.

## Materials and Methods

### Study population and linked electronic records

Anonymised patients were selected from the CALIBER cohort, which includes linked data from: (1) primary care EHR data: diagnoses coded using the Read system by general practitioners during consultation or by practice administrators from hospital discharge letters (from the Clinical Practice Research Datalink) and prescriptions; (2) secondary care administrative records: diagnoses and procedures recorded using the ICD terminology and Office of Population Censuses and Surveys Classification of Interventions and Procedures (OPCS-4, comparable to the American Medical Association Current Procedural Terminology medical classification system) by audit nurses after patient admission by abstracting data from hospital records (from Hospital Episode Statistics); (3) administrative mortality data from death certificates where cause(s) of death are recorded by a doctor and ICD-9 and ICD-10 codes added by trained non-clinical coders (from UK Office of National Statistics; ONS); (4) small-area patient social deprivation information from multiple administrative data sets (from ONS) [12]. CALIBER was approved by the Lewisham Local Research Ethics Committee (ref:09/H0810/16 date: 08/04/2009) and the Ethics and Confidentiality Committee (ECC) (ref: ECC 2-06(b)/2009 CALIBER dataset). CALIBER has been registered with the University College London Data Protection Officer (ref: Z6364106/2009/2/26). CALIBER EHR data are anonymized; individual informed consent was not sought from study participants.

Inclusion criteria were: age greater than 30, minimum one year of validated data prior to entry, and registration at a primary care provider with up-to-standard data. This defined a base cohort of 2,128,151 individuals in which to identify AF. Exclusion criteria were any records of AF diagnosis prior to cohort entry, or the first record of AF after entry being a term indicating monitoring of existing AF or a historical diagnosis of AF. Patients were included and followed-up from the date they met all inclusion criteria or January 1^st^, 1998, whichever was later. Follow-up ended on: the first of the administrative censoring date for primary care data (March 26^th^, 2010); last data collection date for a particular primary care provider; a patient leaving their primary care provider; or patient death as recorded in ONS. Risk factor analyses excluded patients with missing data for blood pressure (BP) measurements, body mass index (BMI), ethnicity or index of multiple deprivation score.

### Strategy for EHR phenotype development

**Figure 1:**
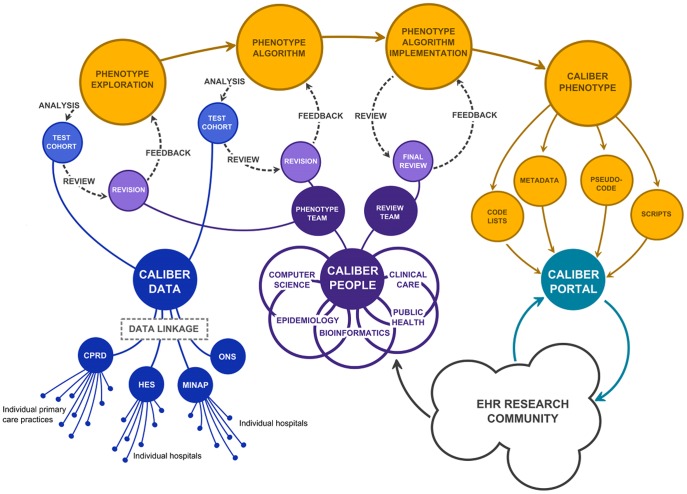
Illustration of phenotype algorithm developing using the Clinical Research Using Linked Bespoke Studies and Electronic health records (CALIBER) programme. CPRD represents the Clinical Practice Research Data link; HES represents Hospital Episode Statistics; MINAP is the Myocardial Ischaemia National Audit Registry; ONS is the UK Office of National Statistics (mortality and social deprivation data).

The CALIBER approach to EHR phenotype development iteratively cycles between expert discussion, review of codes and their semantic relations, and analysis of data (see Figure 1). An initial case definition listing codes, or combinations of codes, indicating diagnosis of a condition is drafted based on discussion with experts in the clinical phenotype, epidemiology, computer science, and bioinformatics. For AF, this preliminary definition only included diagnosis codes directly related to AF from primary or secondary care (extraction of data from free text or image processing is currently limited), but we also identified codes for medications and procedures used in AF treatment for further investigation (lists of all identified codes are available online on the CALIBER Data Portal at www.caliberresearch.org).

#### Initial examination of code usage

A test data set of 100,000 patients was used to investigate how frequently codes were used in practice, and the relationship between diagnosis codes and those for medications and procedures. We found, as have others [53], that although codes for AF subtypes exist within the Read system, they are infrequently used and most patients simply have an all-encompassing diagnosis of AF recorded. Codes indicating an existing condition (e.g. when taking a new patient's history) and monitoring of AF are used, but the main codes recorded relate to a diagnosis of AF. We examined procedure and prescription codes to see if they could assist in identifying additional cases. For procedure codes, the overall frequency was low and they were almost always recorded for patients with a pre-existing AF diagnosis. However, many patients had prescription records for warfarin or digoxin (medications used almost exclusively to treat AF during the time frame of the available data), but no AF diagnosis code.

Review of these results by the expert group led to three decisions: (i) due to the infrequent coding of AF subtypes in primary care, and the single ICD-10 code in secondary care, we should develop an AF phenotype algorithm combining all subtypes; (ii) the case definition should not include procedure codes; (iii) where AF-related prescriptions were made without recorded diagnoses, we should investigate whether a diagnosis of AF could be reasonably inferred. To pursue the latter aim, we developed case definitions based on clinical knowledge of treatment patterns strongly indicative of AF; warfarin prescriptions in the absence of prior deep vein thrombosis (DVT) or pulmonary embolism (PE), or digoxin prescriptions in the absence of heart failure (HF) were taken as evidence to support an “inferred” diagnosis of AF. These conditions were identified using previously defined CALIBER phenotype algorithms for DVT, PE, and HF (see CALIBER Data Portal at www.caliberresearch.org for details).

### Exploration of the EHR

#### Refining disease onset

We investigated the time elapsed between incident AF diagnoses recorded using ICD-10 or Read codes in multiple data sources to see if combing data improved estimation of disease onset. We also investigated the utility of further refining onset using an indicator marker (irregular pulse), examining the frequency of these codes and the time that elapsed between the relevant code(s) being recorded and a subsequent coded diagnosis of AF.

#### Disease case identification

We investigated whether combining multiple sources of EHR data increased the overall number of disease cases identified by permitting us to infer AF diagnoses on the basis of patterns of medication use and comorbid conditions.

### Characteristics of diagnosed patients

As the phenotype algorithm we developed was used to identify diagnoses from different data sources over an extended time period, we wanted to explore whether there were context-level and/or patient-level differences in the cases identified. We quantified the unique and non-unique AF cases identified in each source. We then investigated the relationship between the data source and (i) diagnosis context, specifically the year of diagnosis and whether AF was the primary or secondary reason for admission for secondary care diagnoses (HES provides up 15 secondary diagnosis codes); and (ii) individual patient characteristics at diagnosis including sex, age, and important comorbid conditions (HF, myocardial infarction, hypertension, stroke, diabetes, thyroid disease, renal failure, and chronic obstructive pulmonary disease).

### Association with known risk factors

The face validity of the CALIBER AF phenotype was evaluated by conducting a pre-specified analysis of the association between AF diagnosis and factors for which there is strong prior evidence of association with AF diagnosis from both clinical observations and multiple epidemiological studies: HF, hypertension, and myocardial infarction (MI) [11]. Cause specific Cox proportional hazards models were used to estimate hazard ratio and 95% confidence intervals for incident AF diagnosis associated with baseline measures of risk factors, adjusted for age, sex, and primary care practice [54], [55]. All analyses excluded patients diagnosed with AF prior to study baseline. All statistical analyses were conducted in R version 15.2 for Mac and Linux [56].

## Results

### Sample characteristics

We identified 24 codes (23 Read codes and one ICD-10 code) relating to AF diagnosis. Ten codes refer to monitoring of pre-existing AF, three confirm a prior diagnosis of AF, and the remaining 11 indicate diagnosis by a current care provider. The CALIBER cohort of 2,128,151 participants included 33,383 individuals with an AF code in their record indicating diagnosis prior to cohort entry. Thus, at baseline, approximately 1.6% of the sample had already received a diagnosis of AF, which is similar to prevalence estimate of 2.0% (95% C.I. 1.6–2.4%) provided by the recent UK-based general population ECHOES study [57]. Of the remaining 2,094,768 patients without an AF diagnosis at baseline, 72,793 received their first recorded AF diagnosis code during the study period. A total of 22,939 (45.2%) of patients were initially diagnosed in primary care, with the remaining 39,863 (54.8%) initially diagnosed in secondary care (those diagnosed on the same date in two sources were attributed to secondary care).

### Exploration of the EHR

#### Refining disease onset: Timeframe for diagnosis

To investigate whether combining data from primary and secondary care improved resolution of disease onset, we examined the data for 28,795 individuals with incident diagnoses recorded in both primary and secondary care. The time elapsed between AF diagnoses in the two sources depended on the source of the initial diagnosis. An AF diagnosis was first recorded in primary care for 13,707 individuals, and in secondary care for 10,380 individuals (for 4,708 individuals the dates were the same). The median time from primary care diagnosis to secondary care diagnosis was just over one year (367.0 days), while the mean was almost two years (724.4 days). In contrast, the median time from secondary to primary care AF diagnosis was 20 days (mean 212.6 days).

#### Refining disease onset: Irregular pulse

The primary care Read code system includes five codes for pulse palpation: two indicate a normal or “regular” pulse, and three indicate an “irregular” pulse. Only 1,252 (1.78%) of the 72,793 participants with an incident AF diagnosis had any pulse palpation recorded between study entry and AF diagnosis, with irregular pulse the record closest to AF diagnosis for 964 patients (77.1% of those with any pulse palpation recorded). The median time from first irregular pulse to AF diagnosis in patients where both were recorded after study entry was 71 days. Less than half (40.1%) of patients were diagnosed with AF by 30 days after the irregular pulse code, with 65.8% diagnosed by 12 months.

#### Disease case identification: Inferred diagnosis

Inferred diagnoses were identified based on a combination of 262 codes: 63 relating to prescriptions (36 for warfarin, 27 for digoxin) and 199 excluding conditions (97 for prior HF, 60 for prior DVT, 22 for prior PE) and procedures (20 for heart valve replacement). A total of 39,527 patients met the criteria for an inferred diagnosis of AF during the study period. Warfarin prescriptions accounted for 18,714 (47%) patient diagnoses, digoxin prescriptions for 10,592 (28%), and the remaining 10,221 (26%) had both prescription patterns. A small percentage of patients with an inferred diagnosis (3,754; 9.5%) received coded or historical diagnoses of AF prior to cohort entry. Of the remaining 35,773 individuals, 28,305 (71.6%) had an AF diagnosis code recorded during follow-up and 7,468 (18.9%) had no codes relating to AF diagnosis in their record.

Of the 28,305 individuals who met the inferred AF diagnosis criteria and had an AF diagnosis code within the study window, the majority (75.7%; 21,420 individuals) received the diagnosis code before meeting the criteria for an inferred diagnosis, and for a further 11.2% (3,167 individuals) this occurred on the same day. Thus only 13% of patients (3,718 individuals) met the inferred diagnosis criteria before an AF diagnosis was recorded. For these 3,718 individuals, the average time between an inferred diagnosis and receiving a diagnosis code was 19.8 months (median 6.54 months). Within 30 days of meeting inferred diagnosis criteria, 21.1% of these patients received a diagnosis code; 59.7% received a diagnosis code within one year. However, the temporal relationship between these diagnoses varied depending on the year of the initial inferred diagnosis; the proportion receiving a diagnosis code within 12 months increased gradually over time from 37.1% in 1998 to 92.3% in 2009. The proportion of AF cases based on inferred criteria also decreased over the study period, from just over 15% of cases in 1998 to less than 10% of cases from 2006 onwards.

### EHR phenotype algorithm

The results above informed the development of the AF phenotype algorithm in two ways. First, as pulse palpation was only recorded for a small minority of patients we concluded it did not provide enough additional information to warrant inclusion in our current AF case definition. Second, although examining the pattern of treatments and co-existing conditions did identify additional disease cases, without additional information (e.g. review of free text) we could not confidently conclude that patients without a recorded AF diagnosis code should be considered as cases, or that medication prescriptions represent a diagnosis date. Consequently, we included individuals with only an inferred diagnosis in our EHR case definition as a separate category, and used date of recoded AF diagnosis code in preference to date of meeting inferred criteria.

The case definition for AF using the phenotype algorithm thus had three categories:

1. Historical: first recorded AF code indicates monitoring of an existing condition, or reference to a previous AF diagnosis.
2. Diagnosed: first record is a diagnosis code for AF; preference given to the earliest dated record rather than diagnosis source (i.e. no preference for primary *versus* secondary care).
3. Inferred: no diagnosis code is present, but the patient record includes a warfarin prescription in the absence of prior DVT or PE, or a digoxin prescription in the absence of HF.

**Figure 2:**
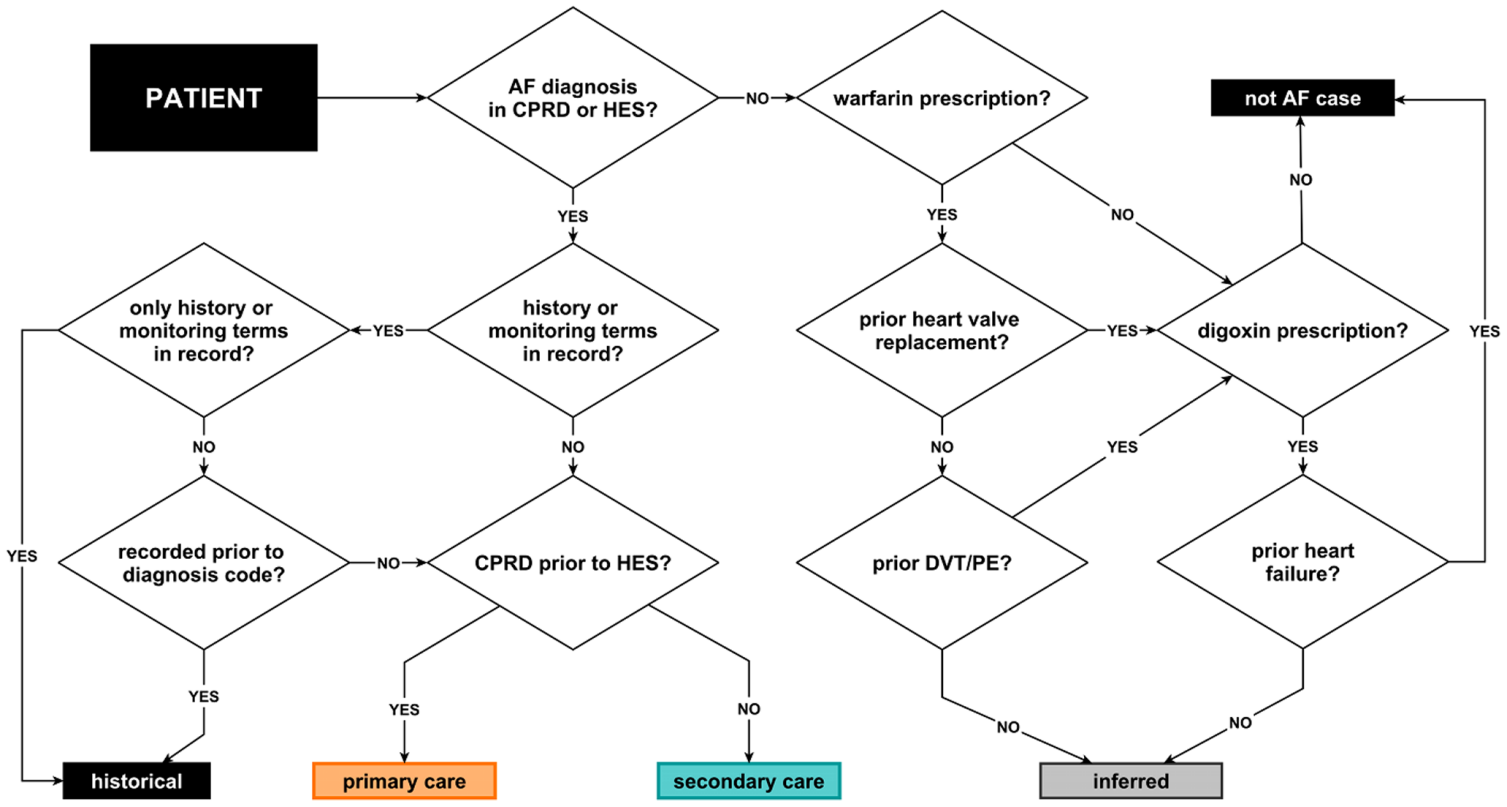
Flow diagram illustrating CALIBER phenotype for atrial fibrillation.

The phenotype algorithm incorporates these definitions in a hierarchical, mutually exclusive manner (see **Figure 2**). If the earliest recorded AF codes relate to a historical diagnosis or monitoring, the patient is in category 1 which precludes inclusion in other categories. If these codes are absent, then the presence of a coded diagnosis from primary or secondary care places a patient in category 2. Finally, in the absence of a coded diagnosis, a patient may be allocated to category 3, depending on the combination of prescriptions and diagnoses in their record. Otherwise a participant is treated as undiagnosed.

### Characteristics of diagnosed patients

**Figure 3:**
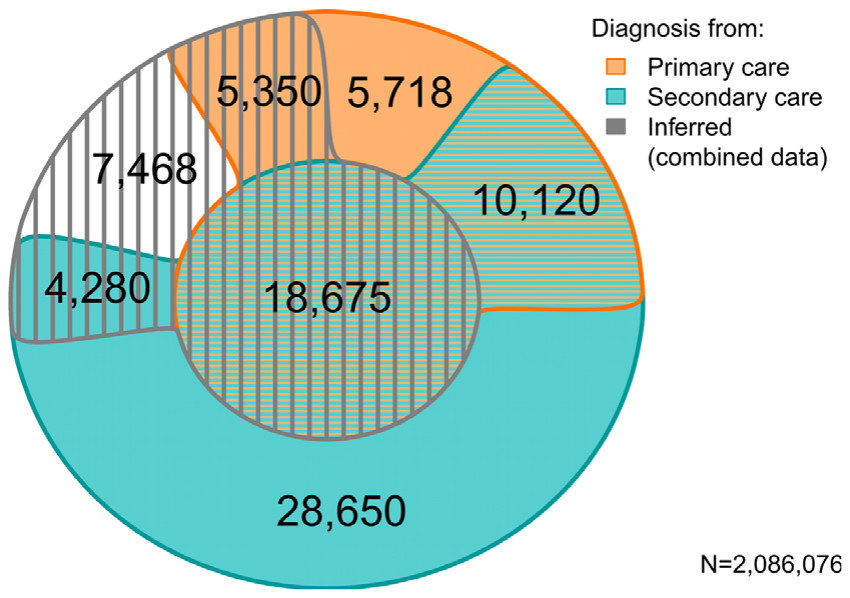
Euler diagram displaying the number of incident cases identified in the different sources, including overlap between multiple sources.

Using the phenotype algorithm we identified 80,261 individuals with an incident coded or inferred AF diagnosis in the CALIBER cohort. Of these, 7,468 had no diagnosis code but met the inferred diagnosis criteria. Almost half the patients with a diagnosis code (39.6%; 28,795 individuals) had diagnoses recorded in both primary and secondary care (see **Figure 3**). All sources provided unique diagnoses, but substantially more were identified from secondary care, which provided almost three times the number of unique cases (32,930 cases compared to 11,068 from primary care). The proportion of AF cases identified in primary care or by inferred diagnosis decreased by year of diagnosis, whereas the proportion identified in secondary care increased, but no threshold effect was identified around the introduction of the QOF in 2004.

**Figure 4:**
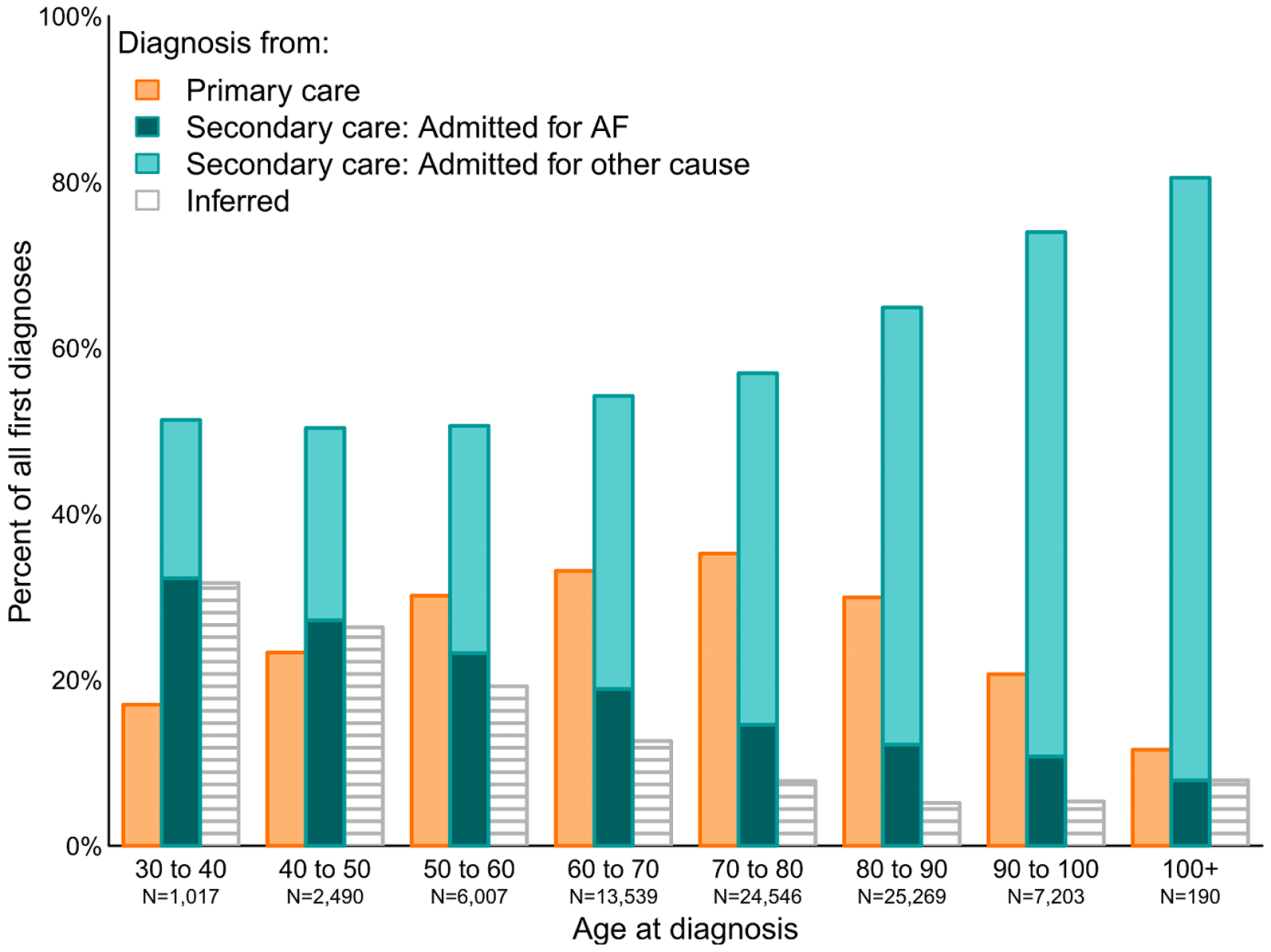
Proportion of incident atrial fibrillation cases identified in each source by age at diagnosis.

The proportion of cases contributed by each source differed by age at diagnosis; individuals identified by inferred diagnosis criteria made up a greater proportion of cases diagnosed at younger ages (≤60 years), while cases diagnosed at older ages (≥80 years) were mostly identified from secondary care data (see **Figure 4**). The proportion of cases identified in primary care was highest for ages 60–80 years, but for all age groups primary care contributed fewer cases than secondary care. For patients diagnosed in secondary care, AF was more likely to be the main diagnosis for the hospital episode when individuals were younger (≤50 years), whereas amongst those diagnosed at older ages AF was much more likely to be a secondary diagnosis made during admission for another condition. Patients with diagnoses recorded only in secondary care were slightly more likely to be female compared to those with diagnoses in both data sources, primary care only, or inferred diagnoses (51.3%, 48.2%, 48.8% and 47.6% female respectively).

The percentage of patients with comorbid conditions at the time that their AF diagnosis was recorded differed by source of diagnosis (see **Table 1**). Patients for which an AF diagnosis was drawn only from secondary care were more likely to have already received a diagnosis for all the conditions examined, with the exception of hypertension, than those with a diagnosis drawn from primary care or meeting the inferred diagnosis criteria. The difference between data sources was largest for renal failure; the percentage of patients with renal failure amongst those diagnosed with AF in secondary care only was twice that of patients with AF diagnoses recorded in the other data sources (22.4% *versus* 10.9%, 11.0%, and 10.0%). A similar, although less extreme, pattern was also observed for HF, MI, stroke, and diabetes (Type 2). This does not appear to be completely due to differences in the age of patients from each source as even within age groups the percentages of patients with pre-existing conditions were still higher for those diagnosed in secondary care, particularly for HF and diabetes. In contrast, the percentage of AF patients with hypertension was highest amongst those with a primary care diagnosis (86.0% for primary and secondary, 86.2% for primary only, compared to 83% for secondary only and 78% for inferred).

**Table 1:** Percentage of patients with different comorbid conditions at date of atrial fibrillation diagnosis, by source of diagnosis.

Characteristic	Category	Source of diagnosis			
		Secondary only (N = 32930)	Primary and secondary (N = 28795)	Primary only (N = 11068)	Inferred (N = 7468)
HF		18.8	15.1	12.7	8.5
MI		13.2	10.0	8.3	14.1
Stroke		9.2	6.0	6.2	8.7
Diabetes	Type 1	0.62	0.39	0.49	0.90
	Type 2	14.73	10.79	9.40	9.53
	NOS	1.76	1.13	1.38	1.94
Hypertension		83.0	86.0	86.2	78.0
Thyroid disease	Hyper	1.7	1.5	1.6	1.0
	Hypo	8.5	7.1	6.8	5.6
Renal failure		22.4	10.9	11.0	10.0
COPD		46.9	44.7	40.9	38.7

Practice Research HF indicates heart failure, MI indicates myocardial infarction, COPD indicates chronic obstructive pulmonary disease, NOS indicates not otherwise specified. Note that some conditions may have been recorded on the same date as the atrial fibrillation diagnosis.

### Associations with known risk factors

**Figure 5:**
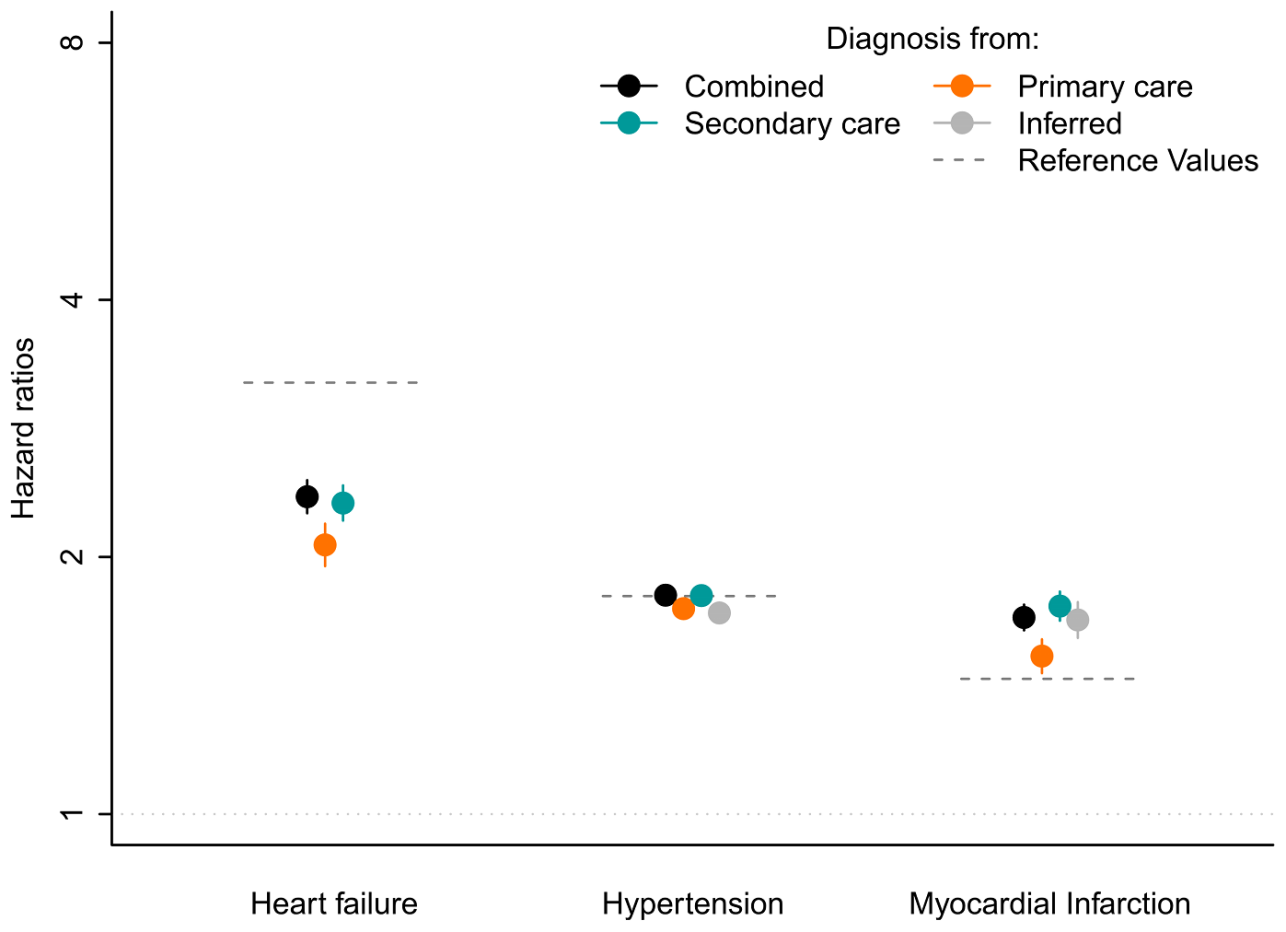
Hazard ratio estimates and 95% confidence intervals for pre-specified risk factors of interest. Results are shown separately for associations between each risk factor and incident AF, defined according to each source of cases and for a composite using all sources. All analyses were adjusted for age, sex, and practice ID. Note that the use of heart failure diagnosis in the algorithm for inferred AF precludes estimation of the hazard ratio. The dashed lines are point estimates of hazard ratios from the Framingham Heart Study for the same risk factors, adjusted for age and sex (see reference [58]).

The associations between pre-specified risk factors and incident AF were consistent in magnitude across EHR sources and with estimates from traditional consented cohorts (see **Figure 5** and **Table S1**). For HF, the hazard ratio estimate was 2.07 (95% CI 1.95–2.19) using only primary care data for AF diagnosis, 2.31 (2.21–2.43) for secondary care data only, and 2.35 (2.25–2.46) for both sources combined (an inferred diagnosis could not be used for the HF analysis as this diagnosis is incorporated into the case definition). For hypertension, the hazard ratio estimates were 1.74 (95% CI 1.70–1.78) for primary care only, 1.80 (1.76–1.84) for secondary care only, 1.72 (1.68–1.77) for inferred diagnoses, and 1.80 (1.77–1.84) for the composite endpoint. The hazard ratio estimates for MI were 1.53 (1.46–1.60) for primary care only, 1.75 (1.68–1.82) for secondary care only, 1.69 (1.61–1.77) for inferred diagnoses, and 1.70 (1.64–1.76) for the composite endpoint.

The estimates for hypertension and MI were comparable to age and sex adjusted results from traditional cohort studies such as the Framingham Heart Study [58] (HR 1.80, 95% C.I. 1.48–2.18 for hypertension; HR 1.44, 95% C.I. 1.02–2.03 for MI) as well as those from the other EHR studies (see **Table S1**). The estimates for heart failure were towards the lower bound of those obtained from the Framingham Heart Study (HR 3.2, 95% C.I. 1.99–5.16) and EHR studies.

## Discussion

We explored the characteristics of the information recorded around the diagnosis of a chronic condition, AF, in multiple linked data sources for a cohort of 2,128,151 individuals from the general population. This exploration highlighted a number of key findings with implications for EHR research on AF, and on chronic conditions more broadly. We found that: (i) refining the timing of disease onset can potentially be improved by the clinically-informed use of data that goes beyond diagnosis codes for the condition in question, but what is recorded as part of routine clinical practice may differ substantially from clinical guidelines; (ii) integrating data from multiple EHR sources and administrative data does improve case detection; (iii) the context in which data are collected may have an impact on the characteristics of the disease cases identified. We used this information to develop an EHR phenotype for identifying AF disease cases that was informed by an understanding of the patient record, and evaluated the face validity of it using epidemiological analyses.

Combining data from multiple sources to identify AF cases helped to refine estimates of disease onset in this sample. Using primary care data brought the date of diagnosis forward by one to two years for patients subsequently diagnosed in secondary care. Although there was a slight lag (median 20 days) from a diagnosis being recorded in secondary care to it being recorded in primary care, this more likely indicates a delay in transfer of diagnosis information from hospital to general practitioner than separate diagnoses. Pulse palpation records were investigated because this is recommended as a screening test for AF in primary care [11], [14]. In our cohort, very few AF patients (just over 2%) had a record of pulse palpation prior to diagnosis and therefore these data had limited use for refining disease onset and were not incorporated into the phenotype algorithm. This underlines the importance of understanding the EHR; a phenotype algorithm for AF based only on clinical guidelines could have required pulse palpation prior to diagnosis and would have excluded the vast majority of cases drawn from primary care data. However, Nicholson *et al*., [2] found that “indicator markers” for rheumatoid arthritis in primary care, such as joint signs and symptoms or non-steroidal anti-inflammatory drug prescriptions, were potentially informative so the utility of “indicator markers” and screening tests should be evaluated on a disease-by-disease basis, and may inform further quality of care research as well as EHR research.

Integrating data from multiple sources identified more AF cases than examining any single source, as has been demonstrated for other cardiovascular conditions [7], [59]. This was primarily due to the fact that a substantial percentage of cases were unique to each data source (13.8% from primary care, 41.0% from secondary care), but integrating data from both sources to infer diagnoses also identified unique cases (9.3%). This demonstrates that clinically informed combinations of treatment records and diagnoses of other conditions can be useful for interrogating EHR datasets, although this may not be true for all conditions in all contexts. For example, Pascoe *et al.* [60] found that procedure codes (such as mastectomy) and prescriptions (such as tamoxifen) could be combined to improve identification of cancer cases in UK EHR data, but we found that procedure codes (such as direct current cardioversion) did not improve AF case detection because they were almost exclusively recorded in patients with a pre-existing AF diagnosis. Additionally, coding combinations could be so complex for some conditions that this approach is infeasible; the inferred AF diagnosis incorporated not only medications but also whether diagnoses were recorded for another condition for which these medications might be used, namely HF, DVT, and PE. The CALIBER programme facilitated this as case definitions and associated EHR phenotypes had already been developed for these conditions; without access to a resource such as this, use of treatment and/or comorbidity information could be substantially more onerous.

The AF patients we identified differed by data source in regard to age, sex, and comorbid conditions, and also over time. Patients identified in secondary care were comparatively older than those identified in primary care, and in many cases AF was a secondary diagnosis made when the patient was admitted for another condition. These patients were also more likely to have a comorbid diagnosis for another condition such as HF, renal failure, or diabetes. Focusing on only one source of data could, therefore, give misleading results about the age distribution and relative health of the AF patient population; integrating data from multiple sources is important for obtaining a representative sample. Ignoring the temporal context of EHR data could also misrepresent the sample and present challenges for phenotyping. We found, as have others (e.g. [61]), that the impact of clinical guidelines can be investigated using EHR data. In our sample, the proportion of inferred AF cases decreased over the study period, as did the time between meeting the inferred criteria and receiving an AF diagnosis code. This gradual change in diagnostic and coding practices may be due to increasing awareness of AF as a condition warranting specific treatment, and potentially the inclusion in the QOF from 2004, although we did not observe a sharp alteration around this time point. This has broader implications for identification of disease cases in EHR data, particularly where treatment information is incorporated; case definitions and phenotype algorithms may need to allow for temporal changes in clinical practice and recording, and not rely on a single strategy being equally effective for all time points.

### Limitations

The work described here has three major limitations relating to the data sources available for use, the strategy used for the AF case definition, and the capacity for external validation. Currently our phenotype algorithm does not use natural language processing (NLP) or imaging data. Use of non-coded data via NLP has been shown to improve detection of other cardiovascular conditions that are difficult to diagnose, such as angina pectoris [62], but although work in this area for application to CALIBER data is ongoing [63], [64], it is not currently ready for general use. Ideally, AF cases identified in EHR data would be confirmed by electrocardiogram readings that displayed variability in the R-R intervals [14], but this source of data is also not currently available on a national scale.

As our aim was to develop an AF phenotype that was of use to all researchers using EHR data, regardless of computational resources, we did not employ some of the more sophisticated techniques used in some other EHR phenotyping studies. We interpreted the first diagnosis code in a patient record as a confirmed diagnosis, but in other EHR phenotypes researchers have required multiple diagnosis codes (e.g. [65]), or used more complex analytical methods (e.g. [66]). These strategies are undoubtedly useful for EHR phenotyping, particularly if the probability of false diagnoses is high, but this will be disease- and context-specific. In the case of AF in the UK during the time period considered, under-diagnosis is the more likely clinical scenario [16], [17], and thus we deemed one AF diagnosis code sufficient. The inferred AF case definition was developed to capture some patients without a recorded diagnosis, but of course this cannot capture patients for whom no AF-related codes were recorded.

An important aspect of EHR phenotype development is validation, preferably against a “gold standard” (such as a manual review of case notes). We could not validate the AF phenotype in this manner as for the CALIBER programme, the initiation and funding of a separate study is required for re-contacting participants or clinicians to confirm diagnoses or review records. However, previous research has shown that AF diagnoses recorded in NHS primary care have a high degree of reliability even when relying on a single diagnosis code [24], although similar information is not available for secondary care (particularly when AF is not the primary diagnosis). The inferred diagnosis category also requires further validation work, particularly as it incorporates information on multiple diagnoses which may have their own limitations (for example, some HF patients will inadvertently be classified as AF patients if the sensitivity of HF diagnoses is less than perfect). In the near future we will apply CALIBER phenotype algorithms to data from the UK Biobank resource, which provides scope for validation of EHR phenotypes against self-reported data and clinical notes [67]. In the absence of external confirmation of AF diagnoses, we evaluated our phenotype definition by conducting epidemiological analyses of the association between known risk factors for AF onset and disease diagnosis in the CALIBER data set, and comparing our estimates to those from other studies. Our point estimates for the hazard ratios for AF and HF, hypertension, and MI were in the same direction as those obtained from comparable analyses in both traditional cohort [58], [68] and EHR studies [26], [31], which suggests that our AF identification strategy indexes a similar AF patient population.

### Future research

The AF phenotype we have developed has been primarily informed by clinical understanding and interpretation of the EHR data. However, research on EHR phenotypes for other conditions has shown that data-driven approaches, such as using lagged linear correlations, can inform the EHR phenotyping process and facilitate the identification of patient subgroupings [4], [69]. Once more sources of data, such as electrocardiogram results and clinical notes, are available on a national scale such approaches may prove useful for improved identification (especially refining the inferred diagnosis category) and further classification of AF patients.

### Conclusion

Overall, we have developed a transparent and reproducible method for identifying AF cases in data from linked EHR sources that detects more cases than using a single data source. We have also highlighted the importance of exploring the patient record prior to developing EHR phenotype algorithms, including a number of challenges that may be encountered and potential strategies for overcoming them. Development of CALIBER phenotype algorithms is an ongoing, iterative process involving researchers within, and outside, the CALIBER network. To facilitate this, the code lists, case definitions, and algorithm for AF are freely available via from the CALIBER website (www.caliberresearch.org), and we encourage feedback from those who make use of this, and other, CALIBER phenotype algorithms.

## Supporting Information

Table S1 **Hazard ratio point estimates and 95% confidence intervals for selected risk factors and incident atrial fibrillation.**
(PDF)
