# Supplementary material for: Defining Disease Phenotypes Using National Linked Electronic Health Records: A Case Study of Atrial Fibrillation
Source: PLoS One. 2014 Nov 4;9(11):e110900. doi: 10.1371/journal.pone.0110900 (PMC4219705; doi:10.1371/journal.pone.0110900)
Supplement: Table S1 — Hazard ratio point estimates and 95% confidence intervals for selected risk factors and incident atrial fibrillation. (PDF) [file pone.0110900.s001.pdf]

Defining disease phenotypes using national linked electronic health records: a case study of atrial fibrillation

Supporting Information

Table S1. Hazard ratio point estimates and 95% confidence intervals for selected risk factors and incident atrial fibrillation.

**Study**	**Analysis**[d]	**Risk factors**		
		Heart failure	Hypertension	Myocardial infarction
CALIBER[a]	Primary care	2.07 (1.95-2.19)	1.74 (1.70-1.78)	1.53 (1.46-1.60)
	Secondary care	2.31 (2.21-2.43)	1.80 (1.76-1.84)	1.75 (1.68-1.82)
	Inferred	N/A	1.72 (1.68-1.77)	1.69 (1.61-1.77)
	Combined	2.35 (2.25-2.46)	1.80 (1.77-1.84)	1.70 (1.64-1.76)
FHS[b]		3.20 (1.99-5.16)	1.80 (1.48-2.18)	1.44 (1.02-2.03)
MDCS[c]	Women	8.70 (3.60-20.94)	1.74 (1.42-2.13)	1.84 (1.26-2.69)
	Men	4.53 (2.34-8.75)	1.78 (1.48-2.14)	2.03 (1.65-2.49)

[a]ClinicAl disease research using Linked Bespoke studies and Electronic Records.

[b]Framingham Heart Study; Schnabel *et al.* (2009) *Lancet* 373:739-45.

[c]The Malmö Diet and Cancer Study; Smith *et al.* (2010) *Eur J Epidemiol* 25:95-102.

[d]Indicates the type of analysis. For CALIBER, this is the data used to define the incident AF diagnosis; for MDCS these are subgroups.
